# Supplementary material for: Machine Learning Gene Signature to Metastatic ccRCC Based on ceRNA Network
Source: Int J Mol Sci. 2024 Apr 11;25(8):4214. doi: 10.3390/ijms25084214 (PMC11049832; doi:10.3390/ijms25084214)
Supplement: Supplementary file 1 [file ijms-25-04214-s001.zip › FigureS1_Volcanos.pdf]

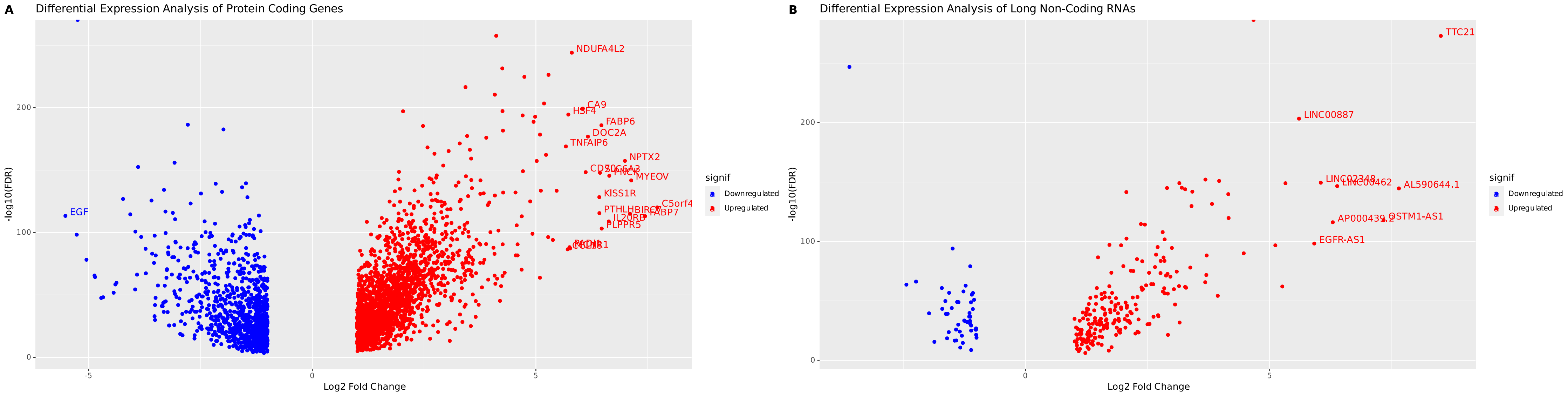

Figure S1: Volcano plots for the differential expressed genes. Volcano plot **A** on top left represents the proteing coding genes differentially expressed, volcano **B** on the top right represents the long non-coding genes differentially expressed. The red genes are upregulared and the blue genes are downregulated.The highlighted are the overexpressed or downexpressed, with a |log2FC|> 5 for teh protein and long non- coding genes.
