# Supplementary material for: Machine Learning Gene Signature to Metastatic ccRCC Based on ceRNA Network
Source: Int J Mol Sci. 2024 Apr 11;25(8):4214. doi: 10.3390/ijms25084214 (PMC11049832; doi:10.3390/ijms25084214)
Supplement: Supplementary file 1 [file ijms-25-04214-s001.zip › FigureS2_ceRNA_Topology.pdf]

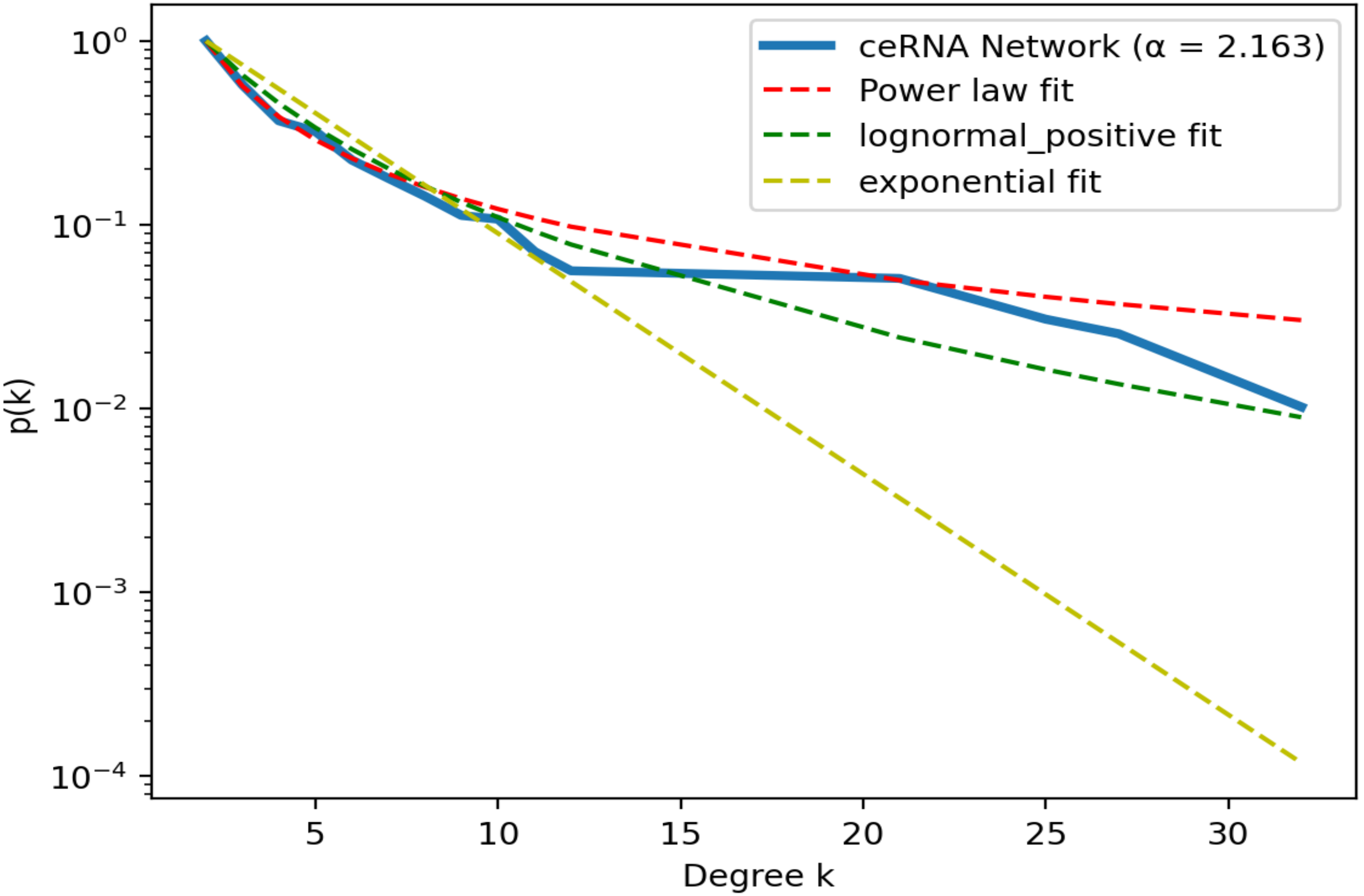

Figure S2: Evaluating the ceRNA network distribuition, comparing with the power-law distribuition (red dashed line), log-normal distribuition (green dashed line), and exponential distribuition (yellow dashed line).
