# Supplementary material for: Machine Learning Gene Signature to Metastatic ccRCC Based on ceRNA Network
Source: Int J Mol Sci. 2024 Apr 11;25(8):4214. doi: 10.3390/ijms25084214 (PMC11049832; doi:10.3390/ijms25084214)
Supplement: Supplementary file 1 [file ijms-25-04214-s001.zip › FigureS3_Multiway_Importance.pdf]

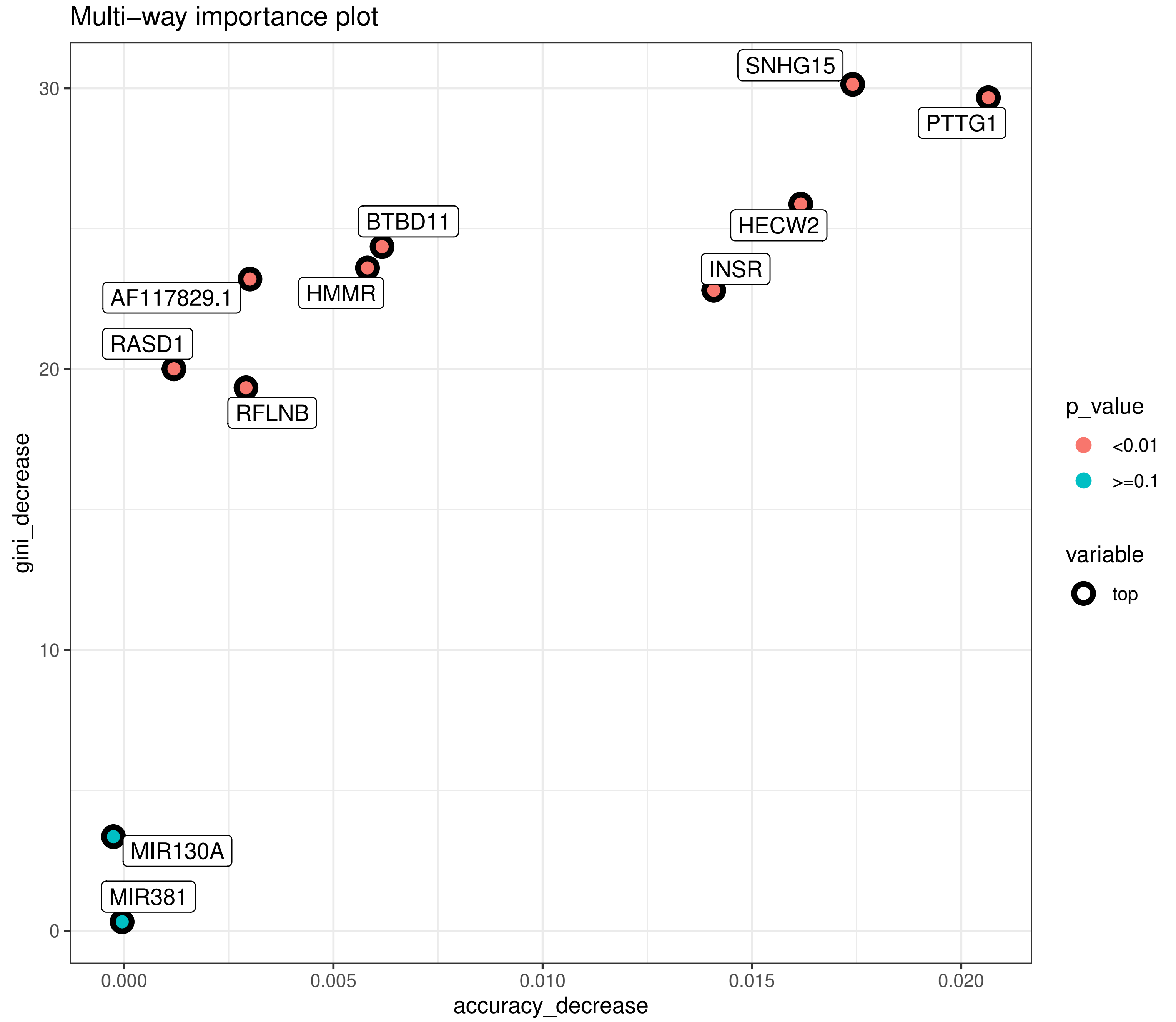

Figure S3: Multi−way importance plot for the 11-gene signature. The mean decrease in accuracy indicates the proportion of samples that become misclassified when the gene of signature is omitted from the model. The Gini Index calculates the raw probability of a sample that is classified incorrectly when selected randomly. MIR130A and MIR381 are alternative symbols for hsa-miR-130a-3p and hsa-miR-381-3p, respectively.
