# Supplementary material for: Machine Learning Gene Signature to Metastatic ccRCC Based on ceRNA Network
Source: Int J Mol Sci. 2024 Apr 11;25(8):4214. doi: 10.3390/ijms25084214 (PMC11049832; doi:10.3390/ijms25084214)
Supplement: Supplementary file 1 [file ijms-25-04214-s001.zip › FigureS4_AUC.pdf]

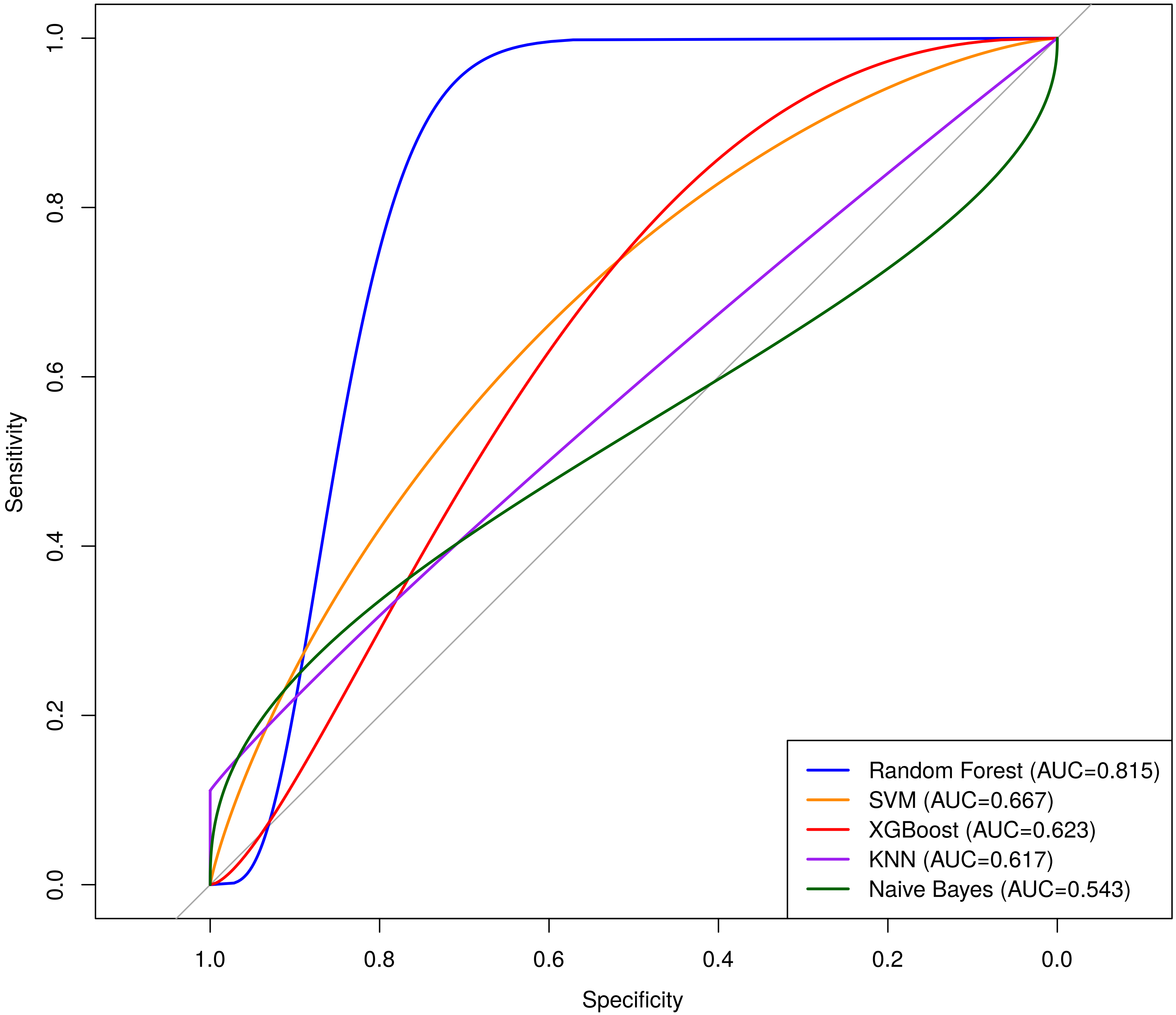

Figure S3: Plot presenting the AUC of the validation process. Where we see a better classification perfomed by the Random Forest method, with an value of 81.5 %
