# Supplementary material for: Machine Learning Gene Signature to Metastatic ccRCC Based on ceRNA Network
Source: Int J Mol Sci. 2024 Apr 11;25(8):4214. doi: 10.3390/ijms25084214 (PMC11049832; doi:10.3390/ijms25084214)
Supplement: Supplementary file 1 [file ijms-25-04214-s001.zip › FigureS5_Kmeans.pdf]

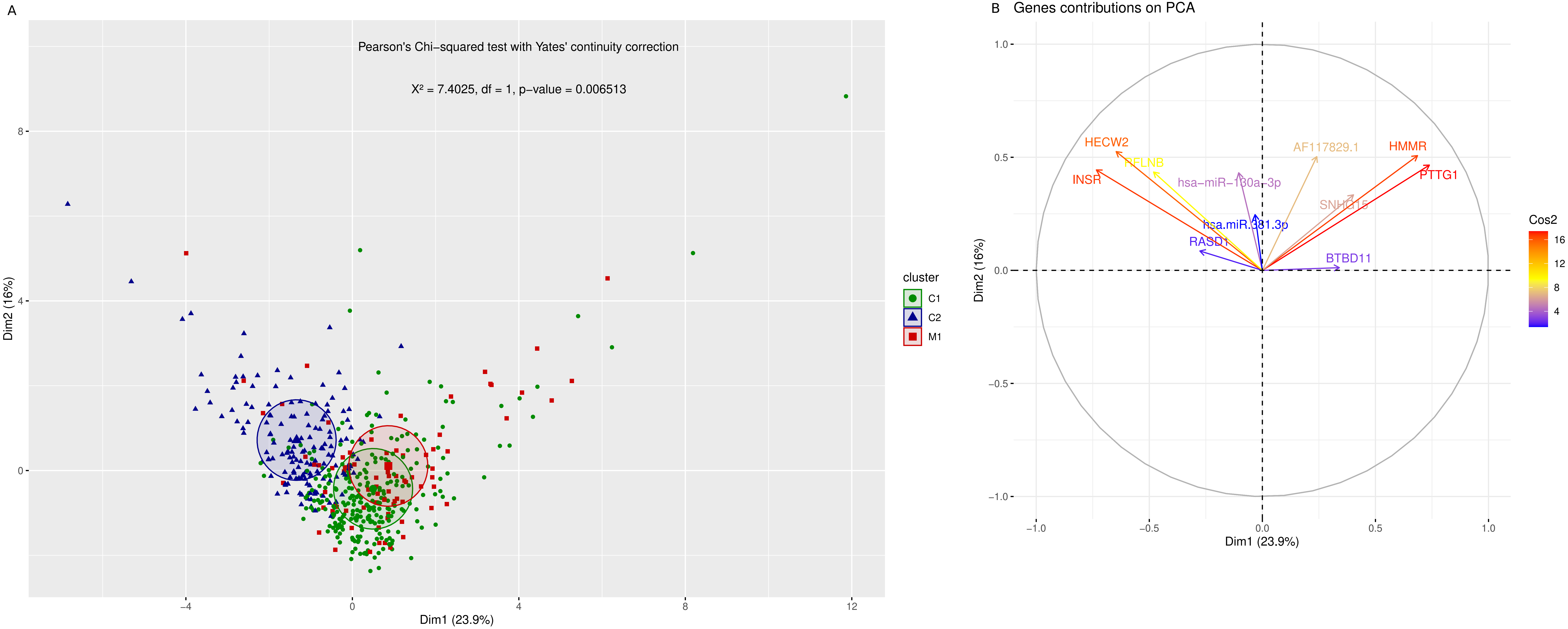

Figure S5: (A) K-means clustering over the TCGA-KIRC samples with the 11-signature genes into two groups (C1 and C2). Subsequently, the dimensional reduction using principal component analysis (PCA), grouped majority of metastastic samples (M1) within the C1 cluster. The chi-squared test revealed a significant association (p-value 0.007) between the metastatic samples and the clustering. (B) Correlation circle pot of gene contributions on PCA. Positively correlated genes are grouped on the positive values of the first and second dimensions (first quadrant), and negatively correlated genes are on the opposite quadrant. The genes with low, mid, and high Cos2 values are coloured in blue, yellow and red, respectively. A high Cos2 indicates a higher contribuition to the specific dimension.
