# Supplementary material for: Machine Learning Gene Signature to Metastatic ccRCC Based on ceRNA Network
Source: Int J Mol Sci. 2024 Apr 11;25(8):4214. doi: 10.3390/ijms25084214 (PMC11049832; doi:10.3390/ijms25084214)
Supplement: Supplementary file 1 [file ijms-25-04214-s001.zip › FigureS6_Oncoplot.pdf]

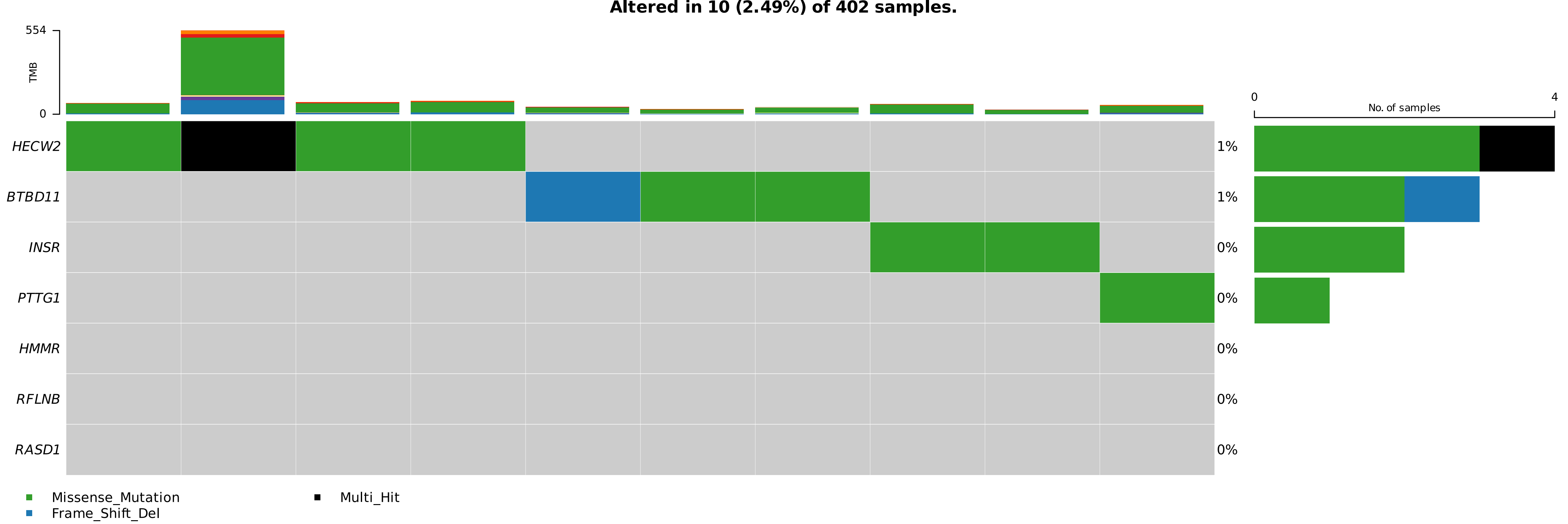

Figure S6: Oncoplot with the mutations recorded in the signature coding genes, The bar graph on the right representes the amount of mutated samples and the bar graph above representes the mutations registeres on these samples
