# Supplementary material for: Machine Learning Gene Signature to Metastatic ccRCC Based on ceRNA Network
Source: Int J Mol Sci. 2024 Apr 11;25(8):4214. doi: 10.3390/ijms25084214 (PMC11049832; doi:10.3390/ijms25084214)
Supplement: Supplementary file 1 [file ijms-25-04214-s001.zip › FigureS7_AalenReg.pdf]

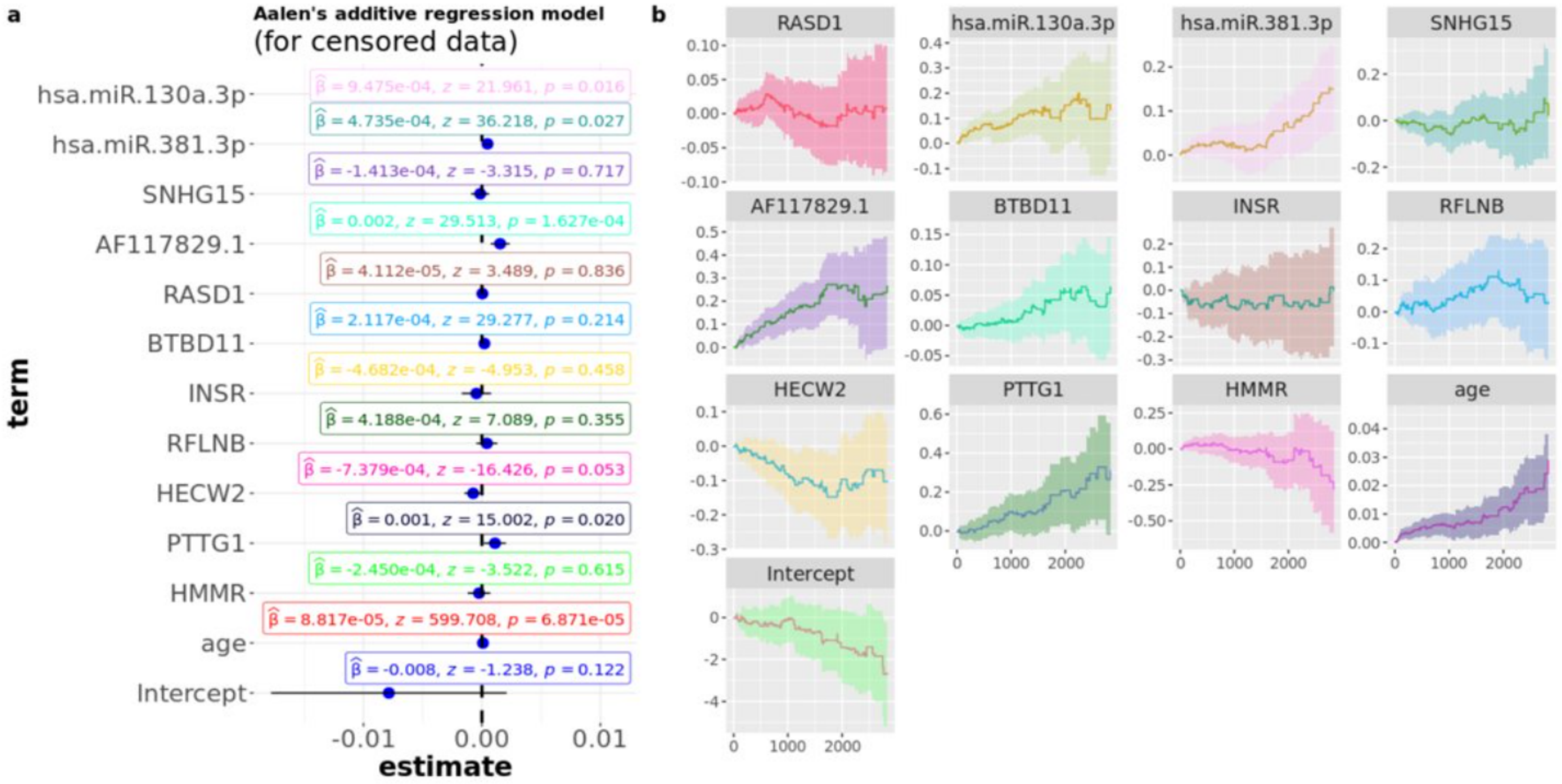

Figure S7: Time-Dependent Hazard Assessment and Covariate Effects. Figure **S7A** represents the covariates coeficients and their p-value associated, and figure **S7B** represents the Hazard Ratio curve, indicating the covariates behavior through time.
