# Supplementary material for: Machine Learning Gene Signature to Metastatic ccRCC Based on ceRNA Network
Source: Int J Mol Sci. 2024 Apr 11;25(8):4214. doi: 10.3390/ijms25084214 (PMC11049832; doi:10.3390/ijms25084214)
Supplement: Supplementary file 1 [file ijms-25-04214-s001.zip › FigureS8_miRNAsEnrichment.pdf]

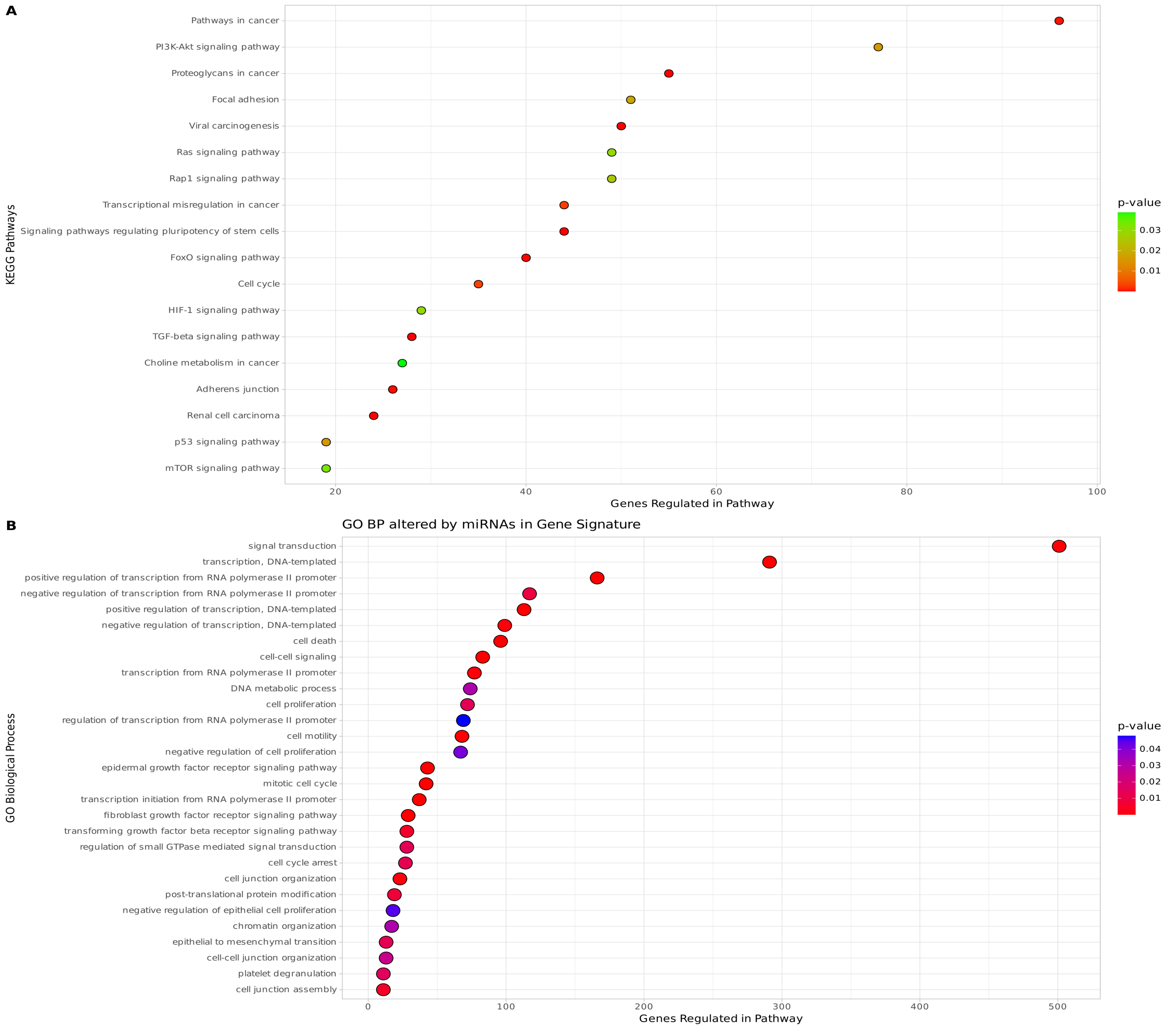

Figure S8: Functional annotation made from (a) KEGG and (b) Gene Ontology using the targets of miRNAs participating in the signature. In both, the Y axis represents the annotated pathways in the databases and the X axis represents the number of genes regulated by the miRNAs in the pathway.
