# Supplementary material for: Machine Learning Gene Signature to Metastatic ccRCC Based on ceRNA Network
Source: Int J Mol Sci. 2024 Apr 11;25(8):4214. doi: 10.3390/ijms25084214 (PMC11049832; doi:10.3390/ijms25084214)
Supplement: Supplementary file 1 [file ijms-25-04214-s001.zip › Supplementary Tables.pdf]

Table S1: Feature selection techniques and application stage.

Method	Technique	Step
Filter	Generalized Linear Model	Feature Selection and Benchmarking
	Linear Discriminant Analysis	Feature Selection
	Akaike Information Criterion	Feature Selection
	eXtreme Gradient Boosting	Benchmarking and Validation
Wrapper	Boruta	Feature Selection
	Recursive Feature Elimination	Feature Selection
	Lasso	Feature Selection
	ElasticNet	Feature Selection
	Support Vector Machine	Benchmarking and Validation
	Naive Bayes	Validation
	k-Nearest Neighbors	Validation
	Random Forest	Benchmarking and Validation

Table S2: Gene-signature chromosomic location

Gene	Chromossomic Location
AF117829.1	8q21.3
BTBD11	12q23.3
HECW2	2q23.3
HMMR	5q34
hsa-miR-130a-3p	11q12.1
hsa-miR-381-3p	14q32.31
INSR	19p13.2
RASD1	17p11.2
PTTG1	5q33.3
RFLNB	17p13.3
SNHG15	7p13
